# A credit scoring model based on the Myers–Briggs type indicator in online peer-to-peer lending

**DOI:** 10.1186/s40854-022-00347-4

**Published:** 2022-05-03

**Authors:** Hyunwoo Woo, So Young Sohn

**Affiliations:** Department of Industrial Engineering, Yonsei University, 134 Shinchon-dong, Seoul, Republic of Korea

**Keywords:** Alternative credit scoring, Behavioral finance, Credit scoring, Locally weighted logistic regression, MBTI, P2P lending, C40, D91, E41, G21, G41

## Abstract

Although psychometric features have been considered for alternative credit scoring, they have not yet been applied to peer-to-peer (P2P) lending because such information is not available on platforms. This study proposed an alternative credit scoring model for P2P lending by extracting typical personality types inferred from the borrowers’ job category. We projected a virtual space of borrowers by using the affinity matrix based on the Myers–Briggs type indicator (MBTI) that fits each job category. Applying the distance in this space to Lending Club data, we used locally weighted logistic regression to vary the coefficients of the variables, which affect loan repayments, with each MBTI type for predicting the default probability. We found that each MBTI type’s credit scoring model has different significant variables. This study provides insights into breakthroughs in developing alternative credit scoring for P2P lending.

**Supplementary Information:**

The online version contains supplementary material available at 10.1186/s40854-022-00347-4.

## Introduction

Alternative credit scoring models have been developed actively based on borrower’s psychometric properties, such as personality or sentiment, besides typically used variables representing individual features. The increased availability of social and psychological information reduces the cost (Akerlof and Kranton 2000; Liberti and Petersen 2019); thus, credit scoring models reflecting individual’s psychometric properties have emerged in the microfinance sector. For example, Visual DNA, a financial technology firm, developed a credit scoring system to identify individual financial characteristics, such as willingness to repay, based on a picture-based psychological test adopted by many countries. Moreover, Entrepreneurial Finance Labs applied common psychometric features of successful entrepreneurs to credit scoring models for predicting an entrepreneur’s repayment pattern. It improved the performance of traditional credit scoring models (Arráiz et al. 2017).

Peer-to-peer (P2P) lending has a good environment to apply this alternative credit scoring model, given that thin filers obtain their loan more easily on the P2P lending platform than traditional finance institutions. Additionally, P2P platform has accumulated abundant data about them. P2P lending is a service that connects private investors directly to loan seekers through an online platform. Borrowers submit their applications for their loan, and lenders read their documents and select reliable borrowers to invest on the online P2P lending platform. With their simple business model of connecting borrowers with lenders through the online platform, P2P lending companies incur a lower transaction cost burden than conventional financial institutions (Guo et al. 2016). The size of global P2P lending was estimated to be around USD 11 billion in 2014 (Belleflamme et al. 2015), and it has doubled every year (Wardrop et al. 2015). Furthermore, P2P lending has been more remarkable since the COVID-19 pandemic because the P2P loan market plays a role in complementing traditional bank lending and giving a chance to borrowers who cannot use the traditional financial services (Nigmonov et al. 2022; Tang 2019), whereas banks reduce the loan size in crisis (Cumming et al. 2021). P2P platforms actively disclose various borrowers’ information, including their personal characteristics and loan purpose, to reduce information asymmetry and help their lenders’ investment (Yan et al. 2015). However, it can be risky for individual lenders to use P2P lending service, unless it uses a reliable credit scoring model (Wang et al. 2015a). Various approaches have been proposed to aid in investors’ decision-making on the P2P platform, using the major platforms’ database, such as Lending Club or Prosper (Wang et al. 2015b).

However, the current literature lacks studies reflecting psychometric properties to credit scoring models in P2P lending, as they have not yet adopted psychometric-related measurements. Many studies generally have only used existing variables when focusing on the prediction accuracy of their models (Florez-Lopez and Ramon-Jeronimo 2015; Li et al. 2021b; Xia et al. 2017) or considering the profitability of their models (Guo et al. 2016; Serrano-Cinca and Gutiérrez-Nieto 2016; Wang et al. 2021). They identified the lack of other new independent variables as a limitation for credit scoring (Volpone et al. 2015). Moreover, studies of psychological information for P2P markets were inconclusive (Wang et al. 2022). Psychological or mental factors of borrowers are highly related to their debt (Fitch et al. 2007); therefore, using additional psychometric variables or replacing existing variables with other psychometric variables in the development of an alternative credit scoring model could be a breakthrough.

This study provides insights into how to derive an alternative credit scoring for P2P lending markets. Despite much data about thin filers in P2P lending markets, previous studies have rarely tried finding new significant variables for explaining them. We infer psychometric properties from the job category of borrowers, an existing but neglected variable (Song et al. 2020), which can represent the borrowers’ aptitude. According to Bradley-Geist and Landis (2012), people within the same occupation category have highly similar personality types. The characteristics of a job can greatly influence an individual’s personality change (Li et al. 2014), and job and business types significantly affect risk propensity (Nicholson et al. 2005). In that sense, the job or occupational variables have often been used for predicting personality or behavioral patterns (King et al. 2017). Therefore, inferring borrowers’ typical tendencies based on their job category could be useful in developing a psychometric credit scoring model.

We proposed a personal psychometric credit scoring model for P2P lending, using the typical personality types of different occupational groups. It identifies how typical tendencies of a borrower’s job affect the fulfillment of the borrower’s obligation. To represent the virtual distance between borrowers’ psychometric properties, we used locally weighted logistic regression (LWLR), unlike typical credit scoring models, that reflects a spatial weight when fitting individual borrower data points. This study obtained spatial weight based on a virtual space of occupational Myers–Briggs’ type indicator (MBTI) personality characteristics. We inferred that the more familiar among the MBTI types, the closer is the distance between those types based on an affinity relationship matrix of the MBTI types. Weights were formed on the basis of this distance, and the samples to be used in the model estimation for individual MBTI type were amplified by extracting them in proportion to each other’s weight. Hence, this model can consider one job and jobs that have a similar tendency to it. This approach is expected to help a credit scoring dealing with the personality, especially for a group of a small number of samples.

We applied our proposed model to Lending Club data. A typical personality type for each occupational group is utilized on the basis of the results of an online MBTI psychological test provided by Truity Psychometrics LLC, a U.S. social psychological research firm known for providing free personality tests with high accuracy. It serves about 30,000 visitors per day in 2020. However, an individual variation might exist within the same job category. Therefore, we used data from borrowers with high job satisfaction, represented by longer job tenure. It assumes that those whose personalities fit their job are pleased to stay in their job because job satisfaction has strong and direct relevance to the career length (Fisher and Herrick 2002). Therefore, only data from borrowers who have been in their jobs for five or more years are used.

This paper is organized as follows. Section 2 summarizes relevant previous studies on credit scoring and MBTI. Section 3 presents the data and method used in the current study. Section 4 shows the results, and Sect. 5 discusses them. Finally, Sect. 6 concludes the paper with a summary.

## Literature review

### Credit scoring with psychometric features for P2P lending

Credit scoring is one of the most important technologies affecting the microfinance sector. This sector has grown rapidly and is regarded as a booming industry. The number of microfinance institutions grew by 474%, with the number of customers rising by 1048% in the 1998–2008 period (Blanco et al. 2013). P2P lending is a microfinance business operating small loans. The P2P lending market has been studied by many researchers, although its platforms are relatively new (Lee and Lee 2012).

Additionally, behavioral characteristics have been adopted in credit scoring models along with typical socio-economic variables. Many factors, such as emotion and social interaction, can affect one’s financial decisions (Oberlechner and Hocking 2004). Prior studies on P2P lending were conducted with various data sources, such as rejected applications of Lending Club (Li et al. 2017; Xia et al. 2018), Chinese P2P lending platform Eloan (Jiang et al. 2018; Xia et al. 2020), social media (Ge et al. 2017), and smartphone data (Ma et al. 2018). Financial researchers have realized the importance of psychological studies on cognitive differences (Muradoglu and Harvey 2012). Human intuition and decision-making biases are primarily related to finance (Kuhnen and Knutson 2011). Many studies have found individual characteristics in demographic variables, such as age, wealth, and occupation (Vissing-Jorgensen 2003; Peress 2004; DaSilva and Giannikos 2006); however, they also appealed to psychological factors for an alternative credit scoring (Jagtiani and Lemieux 2019). It would be more proper to directly adopt psychometric variables (Fernández et al. 2011). Psychology considers the attitudes of market participants, and thus, it is relevant in decision-making research (Oberlechner and Hocking 2004). People with a lack of financial responsibility have identical or similar personality types (Brockett and Golden 2007). Moreover, individuals show their risk inclination as they make purchases with their credit card. By exploiting this information, we can find consumers’ personal characteristics through their MBTI types or Big-five (Saluja et al. 2018).

### MBTI and mechanism of personalities on borrowers’ payment

An individual’s MBTI type is identified by the emotional and reasoning orientations they prefer (Insler et al. 2016). It is based on Jung’s (1923) psychological theory, claiming that certain psychometric preferences conduct certain tasks (Insler et al. 2016). Personality tests are considered tools for identifying various attributes of the personal psychometric profile and character (Walczak and Borkan 2016). Besides being an indicator of personal psychology in research, the MBTI personality test has been widely used in practice (Armstrong et al. 2012), even as a significant predictor of intelligence (Furnham et al. 2007). This theory’s true value is that people can find hidden personal insights through the MBTI personality test (McKenna et al. 2003). Moreover, psychological and behavioral differences are significant factors in understanding the decision-making processes that can explain financial behavior deviating from traditional economic models (Li et al. 2021a; McKenna et al. 2003).

Many studies have shown how individual psychological propensity or MBTI types are highly interrelated with an individual’s job. Accordingly, many studies used a job or occupational variable for predicting personality or behavioral patterns (King et al. 2017). People with similar personality traits and types have similar interests in occupational types (Carless 1999). Moreover, some scholars revealed that people within an occupation have significantly similar personality characteristics (Bradley-Geist and Landis 2012). It means that one’s job has a remarkable effect on the individual differences in personality (Garcia-Sedeñto et al. 2009). On a different note, job and business types are significantly associated with risk propensity (Nicholson et al. 2005).

Some scholars have argued the necessity for further research of psychometric variables in the fields of finance, lending, and credit (Wang et al. 2011). An important relationship exists between the borrower and the repayment of the loan, especially the trouble-free borrowers and the defaulters’ personalities; moreover, the borrowers’ personalities have affected the repayment of the loan (Nyhus and Webley 2001). Many studies have also shown a mechanism of personalities on borrowers’ payment. For instance, Tokunaga (1993) emphasized the predictive power of psychometric variables, showing the tendency for borrowers to reveal a low self-efficacy and an external locus of control and to take little steps to keep their money. This tendency is the way an individual thinks external factors control his/her life. Cognitive dissonance, in which consumers do not feel a sense of cash payment due to consumption through debt, raises the level of the materialism of consumers, and they are likely to show a positive attitude toward having debt (Watson 2003). The lack of self-control consumption style was used to explain indebtedness (Gathergood 2012), and higher impulse leads to more risk and eventually to higher levels of debt (Zuckerman and Kuhlman 2000). A frequency of revolving credit use was negatively related to one’s self-control, self-esteem, self-efficacy, deferring gratification, and locus of control (Wang et al. 2011). It turns out that people who pursue thrill and adventure tend to use small installments more frequently and are more likely to take risks with debts. (Galloway 2002). Moreover, individuals with high compulsiveness are indulgent in spending, such as shopping, because they do not have a strong resistance to temptation, thus having a high frequency of using revolving credit and small installment (Wang et al. 2011).

Although many opinions and attempts positing the necessity of psychological variables for finance have continued to appear, such studies applied to the P2P lending sector are rare despite its abundant public data. In particular, many studies have been conducted with the data from Lending Club, one of the most used P2P lending disclosure data. However, most studies have been carried out without careful consideration of the existing variables in the data. Therefore, it is time for a fresh challenge to further the study based on the borrowers’ data without their psychometric properties. We closely examine the existing variables and derive the derivatives associated with the borrowers’ psychometrics.

## Data and method

### Data source and job–MBTI transformation

This study uses a two-year loan information dataset (2013–2014) provided by Lending Club with R programming, and its code is attached to our supplementary material. The dataset provides credit information for 68,233 borrowers who used Lending Club’s loan service for a 36-month repayment period and are not currently in the process of loan repayment in 2019. The present study only discusses two cases: the loan status of a borrower is “fully paid” within the repayment period or “charged off” for more than 120 days after the loan repayment date. The former is considered a good condition with 59,235 borrowers (86.81%), and the latter is considered a poor condition, also called default, with 8998 borrowers (13.19%).

In the data from the Lending Club, the MBTI personality type of each borrower is not available; therefore, their current job is used as alternative information. We assume that a person whose personality fits with their job tends to stay longer in their job (Fisher and Herrick 2002). Following the 2016 statistics from the Bureau of Labor Statistics that showed that ordinary employees work for at least 3 years, we conservatively select only borrowers whose length of service at the job is 5 or more years. Many previous studies linking career and interest to personality have been going on for a long time before (Hogan and Blake 1999), and Holland (1973) revealed individual personality based on the premise of preference or interest in a particular job.

This study refers to the recommended careers based on MBTI psychological types provided by Truity Psychometrics LLC. The company introduces suitable careers, which are recommended for each MBTI type. The MBTI psychological test data are used to determine the impact of personality types by the occupational group on the degree of loan repayment performance. The MBTI psychological test distinguishes an individual’s personality through four opposing personality functions, also known as a dichotomous preference scale. Each of the four opposing personality functions is divided into two characteristics: (1) Extraversion (E) versus Introversion (I), (2) Sensing (S) versus Intuition (N), (3) Thinking (T) versus Feeling (F), and (4) Judging (J) versus Perceiving (P). One of two characteristics is adopted for each function, resulting in a total of 2^4^ MBTI psychological types.

However, they do not cover all the job categories listed in the Lending Club. Thus, we match borrowers’ job titles with those recommended for the corresponding MBTI type by Truity Psychometrics LLC based on $Sim_{j - w} \left[ {s_{1} ,s_{2} } \right]$, Jaro–Winkler word similarity between string $s_{1}$ and $s_{2}$:

1 $$Sim_{j - w} \left[ {s_{1} ,s_{2} } \right] = Sim_{j} \left[ {s_{1} ,s_{2} } \right] + lp\left( {1 - Sim_{j} \left[ {s_{1} ,s_{2} } \right]} \right)$$

where *l* is the length of common prefixes at the beginning of the strings up to a maximum of four characters, and *p* is a constant scaling factor that indicates the score given for having common prefixes. We set *p* to 0.1, which is commonly used as a standard value. $Sim_{j} \left[ {s_{1} ,s_{2} } \right]$ is Jaro word similarity between strings $s_{1}$ and $s_{2}$ defined as:

2 $$Sim_{j} \left[ {s_{1} ,s_{2} } \right] = \left\{ {\begin{array}{*{20}l} {0,} \hfill & {m = 0} \hfill \\ {\frac{1}{3}\left\{ {m\left( {\frac{1}{{\left| {s_{1} } \right|}} + \frac{1}{{\left| {s_{2} } \right|}}} \right) + \frac{m - t}{m}} \right\}}, \hfill & {m > 0} \hfill \\ \end{array} } \right.$$

where $\left| s \right|$ is the length of string $s$, *m* is the number of matching characters between two strings, and *t* is half the number of characters common between the two strings but have different locations. In matching personal or entity names, the Jaro–Winkler word similarity is widely used and is known as a high-performing method (Cohen et al. 2003; Manaf et al. 2019). Considering the accuracy of matching between job titles, we select only borrowers with a similarity exceeding three quarters.

### Variable setting

The Lending Club dataset has 145 variables, representing the borrower’s information, including loan status, annual income, the number of inquiries in the past six months, current job at the time of loan application, and the number of years of continuous service at work. We remove variables with noise and redundancy (Kou et al. 2021). Then, we select the following (Table 5):

1. variables that had high importance values or significant coefficients in the existing literature (Emekter et al. 2015; Guiso et al. 2013; Jin and Zhu 2015; Serrano-Cinca et al. 2015; Song et al. 2020; Zanin 2020): *Loan*, *Income*, *DTI*, *Rev balance*, *Rev util*, *Job*, *Home*, *Purpose*, and *Grade*;
2. variables related to the borrowers’ financial situation: *Tot balance* and *Cred limit*; and
3. the derived variables made by the proportion of those financial situation-related variables: *Loan per Income* and *Tot balance per Income* (Table 1).

**Table 1:** Summary of the selected variables

Type	Variable	Description
Continuous (original)	Loan	Loan amount/1 M ($)
	Income	Annual income/1 M ($)
	DTI	Debt-to-income ratio^a^
	Rev balance	Total credit revolving balance/1 M ($)
	Rev util	Revolving line utilization rate^b^
	Tot balance	Total current balance of all accounts/1 M ($)
	Cred limit	Total high credit limit/1 M ($)
Continuous (derived)	Loan per Income	Loan/income
	Tot balance per Income	Tot balance/income
Categorical	Job	Length of the job service
	Home	Home ownership
	Purpose	Purpose of borrowing
	Grade	Credit grade assigned by Lending Club

^a^Debt-to-income (DTI) ratio is the ratio of borrower’s total monthly debt payments to the total debt obligations, excluding mortgage and the requested Lending Club loan, divided by the borrower’s self-reported monthly income

^b^The amount of credit the borrower is using relative to all available revolving credit

Then, we remove borrowers with outlier values outside 3-sigma of the mean value for each continuous variable and reclassify the level of each categorical variable so that the group of borrowers can be of similar size.

### Locally weighted logistic regression

The framework for credit scoring based on MBTI types by the occupational group consists of three phases. First, 2^4^ MBTI types are represented in a virtual space for occupational MBTI types. The distance between MBTI types in this virtual space is closer as they are similar. The similarity between MBTI types is expressed by affinity values between them (Table 2). It shows ordinal ranking expressed as an integer from 0 to 4. The higher the rank, the closer the relationship. The distance from type *p* to type *q* $D_{p, q}$ is expressed by a value located in row *p* and column *q* in the affinity rank matrix.

**Table 2:** Affinity rank matrix by MBTI types

	ESTJ	ESTP	ESFJ	ESFP	ENTJ	ENTP	ENFJ	ENFP	ISTJ	ISTP	ISFJ	ISFP	INTJ	INTP	INFJ	INFP
ESTJ	0	1	2	2	1	3	3	4	1	2	3	4	2	4	3	4
ESTP	1	0	3	2	2	1	3	2	2	1	4	3	3	4	4	4
ESFJ	1	2	0	1	3	4	2	3	2	3	1	2	4	4	3	4
ESFP	3	1	1	0	4	3	2	2	3	2	2	1	4	4	4	3
ENTJ	1	3	3	4	0	1	2	2	2	3	4	4	1	2	3	4
ENTP	3	2	4	3	1	0	2	1	4	3	4	4	2	1	3	2
ENFJ	3	4	1	3	2	2	0	1	4	4	3	4	2	3	1	2
ENFP	4	3	3	2	2	1	1	0	4	4	4	3	3	2	2	1
ISTJ	1	2	2	3	2	4	4	4	0	1	1	3	2	3	3	4
ISTP	2	1	4	2	4	4	3	3	1	0	2	1	4	2	3	3
ISFJ	2	3	1	2	4	4	2	3	1	3	0	2	4	4	1	3
ISFP	4	2	2	1	4	4	3	2	3	1	1	0	4	3	3	2
INTJ	3	3	4	4	1	2	2	3	1	3	4	4	0	1	2	2
INTP	4	3	4	4	2	1	3	2	3	2	4	3	1	0	2	1
INFJ	4	4	3	4	3	3	1	2	3	4	1	2	2	2	0	1
INFP	4	4	4	3	3	2	2	1	4	3	3	2	2	1	1	0

Second, based on the distance from a particular MBTI type, the relative weight is assigned to borrowers of other MBTI types. For the model to be specific to a particular MBTI type, the similarity between the MBTI types must be reflected by using a similarity weight. Borrowers of other MBTI types are randomly oversampled with replacement to solve the unbalanced problem, depending on the weight representing the relative distance to the corresponding MBTI type. Therefore, the number of borrowers is increased relative to the weight associated with each borrower *i* according to a specific MBTI type. $w_{{\left( {p,i} \right)}}$ is the similarity weight of MBTI type of borrower *i* in relation to MBTI type *p*. We use a k-square function for $w_{{\left( {p,i} \right)}}$, which is the most commonly used kernel density, defined as:

3 $$w_{{\left( {p,i} \right)}} = 1 - \left( {\frac{{D_{{p, m_{i} }} }}{D}} \right)^{k} \;for \;all\;p$$

where k is a hyper-parameter, $D_{{p, m_{i} }}$ is the distance from MBTI type *p* to $m_{i}$, which is an MBTI type of borrower *i*, and *D* is a bandwidth. For the hyper-parameter tuning, we select the optimal value of the bandwidth *D* and *k*, which maximizes the accuracy of our model by grid search, applying a holdout validation to the sample with a stratified sampling based on loan status and MBTI type. We increase the value of *D* from the maximum affinity distance between MBTI types by 2 unit, adjusting the *k* value from 0.5 to 2.0 in 0.5 unit until the weights of the other MBTI types exceed half of that of MBTI type in a model.

Finally, LWLR is performed on the amplified sample (weighted oversample), so that more borrowers with a similar propensity to a particular MBTI type become reflected in the model. The LWLR model is an extended version of the logistic regression model for considering the influence of spatial variation (Cleveland and Devlin 1988). It is a specialized credit scoring model for predicting the “fully paid,” reflecting more similar personalities for each MBTI type. It reflects different coefficients of the model for each location. In this study, we use individual MBTI types to represent locations for individual LWLR, and the following LWLR model formula is used:

4 $$ln\left( {\frac{{P_{i} }}{{1 - P_{i} }}} \right) = \left[ {\beta_{0} \left( {m_{i} } \right) + \mathop \sum \limits_{k = 1}^{l} \beta_{k} \left( {m_{i} } \right)*x_{ki} } \right]$$

where, for a borrower *i*, $P_{i}$ is the probability of non-default (fully paid), $x_{ki}$ is *k*th independent variable, $\beta_{k} \left( {m_{i} } \right)$ is a coefficient for *k*th independent variable of its MBTI type $m_{i}$, and *l* is the total number of variables. We perform bootstrapping on an individual MBTI model to obtain robust coefficient values. Further, we conduct logistic regression (LR) in two ways: (1) LR with the entire train data instead of differentiating among borrowers’ MBTI groups and (2) LR for each MBTI type. The performance of the methods in financial risk management (Kou et al. 2014) must be evaluated; thus, we compare the proposed LWLR models with the corresponding LR models.

## Results

### Sample reflecting MBTI types

Applying the similarity concept with outlier handling, we obtained a total of 55,820 borrowers for our sample shown in Table 3. Among the borrowers with their MBTI information, the MBTI type with the largest group was ISTJ with 18,124 borrowers and the smallest group was INTP, with 397 borrowers. The average default rate of all MBTI types was 12.98%, and each MBTI type had a default rate ranging from 10.33% to 16.59%. Results reveal a significant difference (*p-*value < 0.01) between INFP and INTP, which had the maximum and the minimum average default rate respectively. This supports the argument of Haack et al. (2012), who specified a difference in personal budget usage depending on MBTI types. Table 4 provides the descriptive statistics of variables for our sample, which shows a class imbalance in *Loan status*, a dependent variable. To detect changes in the increasing or decreasing trends of loan repayment probability at specific values in some variables, we added squared values of the variables related to cash: *Loan*, *Income*, and *Tot balance*.

**Table 3:** Borrowers and default rates by MBTI types in Lending Club

MBTI type	No. of borrowers (%)	No. of Borrowers with charged off [default rate (%)]
ESTJ	2679 (4.80)	311 (11.61)
ESTP	1319 (2.36)	165 (12.51)
ESFJ	2335 (4.18)	339 (14.52)
ESFP	1663 (2.98)	209 (12.57)
ENTJ	10,142 (18.17)	1266 (12.48)
ENTP	581 (1.04)	73 (12.56)
ENFJ	4616 (8.27)	560 (12.13)
ENFP	1620 (2.90)	223 (13.77)
ISTJ	18,124 (32.47)	2399 (13.24)
ISTP	1010 (1.81)	110 (10.89)
ISFJ	7041 (12.61)	1001 (14.22)
ISFP	989 (1.77)	136 (13.75)
INTJ	574 (1.03)	65 (11.32)
INTP	397 (0.71)	41 (10.33)
INFJ	2085 (3.74)	317 (15.20)
INFP	645 (1.16)	107 (16.59)
Mean	3489 (6.25)	458 (12.98)
Total	55,820 (100.00)	7322 (13.12)

**Table 4:** Descriptive statistics of selected variables

Variable	Min	Max	Mean	SD	N	Remark
**Dependent variable**						
Loan status						
Charged off					7322	0
Fully paid					48,498	1
**Independent variable**						
Loan	0.001	0.035	0.012	0.007	55,820	
Income	0.007	0.230	0.065	0.029	55,820	
DTI	0.000	0.400	0.179	0.076	55,820	
Rev balance	0.000	0.064	0.013	0.009	55,820	
Rev util	0.000	1.232	0.567	0.217	55,820	
Tot balance	0.000	0.468	0.109	0.100	55,820	
Cred limit	0.001	0.478	0.134	0.109	55,820	
Loan per Income	0.008	0.500	0.200	0.101	55,820	
Tot balance per Income	0.000	10.502	1.673	1.424	55,820	
**Job**						
≥ 10 years					28,288	
< 10 years					27,532	Reference
**Home**						
Own					5086	
Rent					21,389	
Mortgage					29,345	Reference
**Purpose**						
Debt consolidation					33,537	
Credit card					13,815	
Others					8468	Reference
**Grade**						
A					10,406	
B					21,491	
C					14,416	
D or less					9507	Reference

### Locally weighted logistic regression

We conducted 10 repetitions of the holdout validation for our sample, which divided it into train and test data at a ratio of 7:3. To determine the average accuracy of each holdout validation, we applied a bootstrapping technique with 100 times to individual MBTI model, depending on the combination of values *k* and *D*. The optimization based on the average accuracy of the holdout validations (Table 5) yields 6 and 1.5 as the bandwidth *D* and the hyper-parameter of the kernel density function *k*, respectively.

**Table 5:** The average accuracy of LWLR models according to the combination of *k* and *D*

k	D			
	0.5	1.0	1.5	2.0
4	0.615 (0.022)	0.616 (0.023)	0.616 (0.024)	0.616 (0.024)
6	0.617 (0.023)	0.617 (0.024)	0.618 (0.024)	
8	0.617 (0.023)	0.617 (0.024)		
10	0.617 (0.024)			
12	0.617 (0.024)			
14	0.617 (0.024)			
16	0.617 (0.024)			

Values of the table are expressed as means with standard deviations in parentheses

Table 6 presents the results of the 8 of the 16 models by MBTI type based on the general LR model; the eight models were the main models providing significant findings. The complete results, including the remaining models, are shown in Additional file 1: Appendix A. In the general LR model, all the effects of variables except *Tot balance*^2^, *Tot balance per Income*, *Home (Own)*, and *Purpose (Credit card)* were significant. Furthermore, some variables in the general LR model had significant coefficients with the same sign as those in the LWLR models developed for the 16 MBTI types: *DTI*, *Rev balance*, *Tot balance*, *Cred limit*, *Home (Rent)*, and *Grade*. These variables had a significantly robust effect on the probability of borrowers’ repayment regardless of borrowers’ personality types.

**Table 6:** Result of the main models for “fully paid” borrowers

	LR	LWLR							
		ENTJ	ENTP	ENFP	ISTJ	ISFJ	INTJ	INTP	INFP
Intercept	− 0.128^†^ (0.069)	0.088 (0.055)	0.106 (0.210)	0.058 (0.124)	0.029 (0.041)	− 0.041 (0.058)	0.049 (0.190)	0.092 (0.268)	0.044 (0.202)
Loan	− 48.188** (5.834)	− 41.402** (5.073)	− 35.188^†^ (21.132)	− 26.782* (11.017)	− 43.548** (3.804)	− 33.559** (6.083)	− 47.409* (18.823)	− 38.393 (23.516)	− 33.996^†^ (19.484)
Loan^2^	1352.214** (120.367)	1377.359** (101.545)	1224.617** (431.974)	993.159** (240.924)	1356.752** (75.014)	1051.521** (135.141)	1494.990** (410.588)	1276.214* (504.738)	1082.683* (432.668)
Income	9.518** (1.394)	4.885** (1.118)	4.468 (4.553)	3.935 (2.515)	7.476** (0.830)	7.720** (1.201)	5.658 (4.577)	4.820 (5.799)	4.586 (4.473)
Income^2^	− 46.388** (6.105)	− 21.748** (4.024)	− 22.077 (17.547)	− 21.149* (9.915)	− 32.107** (3.23)	− 35.506** (4.613)	− 22.270 (18.444)	− 21.313 (22.402)	− 21.923 (17.670)
DTI	− 2.121** (0.102)	− 3.052** (0.066)	− 3.129** (0.293)	− 2.882** (0.202)	− 2.787** (0.057)	− 2.49** (0.072)	− 3.101** (0.298)	− 3.133** (0.369)	− 3.029** (0.320)
Rev balance	6.682** (0.972)	8.367** (0.681)	7.375* (2.924)	7.280** (1.574)	10.475** (0.506)	9.544** (0.750)	8.506** (2.924)	8.434* (3.732)	8.961** (3.060)
Rev util	− 0.193** (0.039)	0.100** (0.031)	0.115 (0.129)	0.086 (0.067)	− 0.027 (0.019)	− 0.077* (0.032)	0.070 (0.116)	0.078 (0.154)	0.050 (0.132)
Tot balance	− 5.337** (0.740)	− 7.226** (0.589)	− 7.461** (2.323)	− 7.507** (1.489)	− 6.033** (0.435)	− 5.958** (0.723)	− 7.761** (2.550)	− 7.875** (2.940)	− 7.907** (2.198)
Tot balance^2^	1.288 (0.800)	− 0.447 (0.572)	0.086 (2.261)	0.461 (1.604)	0.265 (0.426)	1.047 (0.773)	− 0.642 (2.709)	− 0.553 (3.025)	− 0.442 (2.568)
Cred limit	5.141** (0.572)	6.858** (0.420)	6.966** (1.788)	6.975** (1.137)	5.296** (0.301)	5.146** (0.505)	7.224** (1.699)	7.375** (2.285)	7.575** (1.589)
Loan per Income	− 0.720** (0.211)	− 1.142** (0.175)	− 1.288^†^ (0.669)	− 1.322** (0.351)	− 1.124** (0.131)	− 1.143** (0.186)	− 1.007^†^ (0.601)	− 1.184 (0.793)	− 1.143^†^ (0.610)
Tot balance per Income	− 0.008 (0.016)	0.020† (0.010)	0.021 (0.046)	0.019 (0.025)	0.027** (0.007)	0.021 (0.013)	0.038 (0.043)	0.034 (0.051)	0.025 (0.041)
Job									
≥ 10 years	0.032* (0.015)	0.004 (0.010)	− 0.001 (0.041)	0.005 (0.026)	− 0.002 (0.007)	0.008 (0.011)	0.003 (0.045)	− 0.006 (0.046)	0.007 (0.042)
Home									
Own	− 0.026 (0.027)	− 0.121** (0.019)	− 0.102 (0.078)	− 0.114* (0.047)	− 0.160** (0.012)	− 0.150** (0.020)	− 0.097 (0.070)	− 0.124 (0.096)	− 0.124 (0.076)
Rent	− 0.213** (0.020)	− 0.300** (0.014)	− 0.284** (0.060)	− 0.257** (0.037)	− 0.303** (0.009)	− 0.255** (0.017)	− 0.284** (0.055)	− 0.301** (0.071)	− 0.267** (0.055)
Purpose									
Debt consolidation	0.037^†^ (0.020)	0.003 (0.014)	0.024 (0.064)	0.013 (0.035)	0.016† (0.009)	0.016 (0.017)	0.026 (0.057)	0.021 (0.064)	0.032 (0.065)
Credit card	0.027 (0.026)	− 0.006 (0.017)	0.004 (0.067)	− 0.019 (0.039)	0.014 (0.012)	− 0.008 (0.021)	0.023 (0.061)	0.024 (0.085)	0.021 (0.071)
Grade									
A	1.342** (0.029)	1.368** (0.019)	1.352** (0.074)	1.359** (0.042)	1.375** (0.015)	1.372** (0.024)	1.350** (0.088)	1.344** (0.093)	1.324** (0.089)
B	0.816** (0.023)	0.823** (0.014)	0.810** (0.056)	0.801** (0.032)	0.836** (0.012)	0.803** (0.016)	0.814** (0.057)	0.808** (0.068)	0.795** (0.057)
C	0.386** (0.021)	0.408** (0.013)	0.402** (0.063)	0.388** (0.029)	0.417** (0.010)	0.394** (0.018)	0.400** (0.060)	0.398** (0.059)	0.393** (0.064)
*N*	96,996	202,968	11,606	31,936	359,604	138,118	11,630	8,130	12,290

Values of the table are expressed as coefficient estimates with standard errors in parentheses

Significance levels at ^†^*p* < .10, *p < .05, and **p < .01

In all models, the effect of *Tot balance* was significantly negative, but that of its quadratic term was not significant. Meanwhile, *Loan* and *Income,* including their quadratic terms, had significant coefficients in both general LR model and most LWLR models, meaning that each of their effects on borrowers’ repayment was reversed when crossing its certain thresholds. From the variable *Loan* with its negative coefficient, the more money borrowers borrowed up to a certain threshold, the less likely they were to pay it off. However, if they borrowed more than this threshold, the opposite occurred. Conversely, the coefficients of *Income* showed the opposite sign of those of *Loan*. The relationship between the loan amount and the annual income through the derivative variable, *Loan per Income*, has a significantly negative coefficient in the models except INTP. Regarding this relationship, borrowers were worse able to pay back their loans as they borrowed larger loan amounts relative to their annual income.

We found that the LWLR models for INTP and ENFP types have no significant trend change in the effect of loan amount and annual income, respectively, which are different from most other models with thresholds for the significant trend change in those variables’ effect. INTP borrowers with a larger loan were more likely to pay it off in that *Loan* it has an insignificant coefficient, but its quadratic term has a significantly negative one in the LWLR model for INTP type. Conversely, ENFP borrowers with a higher annual income were less likely to repay their loan in that the coefficient of *Income* is insignificant, but that of its quadratic term is significantly negative in the corresponding LWLR model.

In the LWLR models for ENTJ, ISFJ, and ISTJ types, the coefficients of some variables that are not significant in those for other MBTI types are significant for each of them: *Rev util* with ENTJ and ISFJ, *Tot balance per Income* with ENTJ and ISTJ, and *Purpose (Debt consolidation)* with ISTJ. Meanwhile, the coefficients of *Rev util* are significant in the models for both ENTJ and ISFJ, with the former positive and the latter negative. The effects of *Tot balance per Income* are significantly positive in the models for both ENTJ and ISTJ. Additionally, among all LWLR models, only the ISTJ type model has significantly positive coefficient of *Purpose (Debt consolidation)*. This means that ISTJ borrowers aiming for their debt consolidation were more likely to pay back their loan than those with other purposes (reference).

Table 7 shows the accuracy, sensitivity, F1-score, and area under the receiver operating characteristics (AUC) of each model to compare the performance of the LWLR models with those of the general LR models fitted to the total sample and each MBTI type. The LWLR models had better average performance than the LR models fitted to each corresponding MBTI type. Moreover, note that although the number of borrowers for INTP type is 397, which is the smallest number of samples, the LWLR model for INTP type definitely showed higher performance in the sensitivity than LR fitted to that type. Sensitivity is an important issue in P2P lending because finding potential default borrowers is more critical than finding fully paid ones in that field.

**Table 7:** Performance of the models for “fully paid” borrowers

MBTI type	Accuracy			Sensitivity (recall)			F1 − score			AUC		
	LWLR	LR	LR for each MBTI type	LWLR	LR	LR for each MBTI type	LWLR	LR	LR for each MBTI type	LWLR	LR	LR for each MBTI type
ESTJ	0.633	0.633	0.616	0.622	0.630	0.611	0.282	0.285	0.269	0.681	0.682	0.663
ESTP	0.605	0.591	0.638	0.681	0.683	0.532	0.299	0.293	0.267	0.680	0.678	0.636
ESFJ	0.601	0.595	0.600	0.621	0.638	0.601	0.310	0.313	0.302	0.644	0.644	0.639
ESFP	0.616	0.608	0.602	0.614	0.635	0.570	0.284	0.287	0.263	0.652	0.656	0.619
ENTJ	0.641	0.633	0.625	0.593	0.609	0.611	0.291	0.292	0.289	0.668	0.672	0.669
ENTP	0.609	0.600	0.598	0.657	0.667	0.605	0.291	0.289	0.266	0.689	0.689	0.644
ENFJ	0.613	0.607	0.582	0.582	0.593	0.575	0.267	0.268	0.250	0.644	0.648	0.626
ENFP	0.606	0.604	0.595	0.597	0.621	0.542	0.292	0.299	0.266	0.635	0.642	0.601
ISTJ	0.621	0.611	0.609	0.617	0.634	0.631	0.301	0.301	0.300	0.662	0.663	0.660
ISTP	0.670	0.657	0.646	0.619	0.638	0.571	0.289	0.288	0.258	0.719	0.722	0.673
ISFJ	0.589	0.587	0.601	0.629	0.640	0.613	0.303	0.306	0.304	0.652	0.653	0.645
ISFP	0.623	0.621	0.607	0.643	0.667	0.532	0.316	0.323	0.268	0.678	0.686	0.615
INTJ	0.636	0.626	0.636	0.637	0.655	0.547	0.281	0.281	0.251	0.662	0.661	0.636
INTP	0.587	0.581	0.661	0.737	0.749	0.483	0.267	0.267	0.225	0.710	0.720	0.628
INFJ	0.595	0.598	0.562	0.582	0.603	0.626	0.304	0.313	0.303	0.642	0.646	0.623
INFP	0.635	0.634	0.588	0.585	0.600	0.543	0.347	0.352	0.303	0.661	0.666	0.600
Mean	0.618	0.612	0.610	0.626	0.641	0.575	0.295	0.297	0.274	0.667	0.671	0.636
Max	0.670	0.657	0.661	0.737	0.749	0.631	0.347	0.352	0.304	0.719	0.722	0.673
Min	0.587	0.581	0.562	0.582	0.593	0.483	0.267	0.267	0.225	0.635	0.642	0.600
SD	0.022	0.021	0.025	0.040	0.038	0.042	0.020	0.021	0.024	0.024	0.025	0.022

## Discussion

**Fig. 1:**
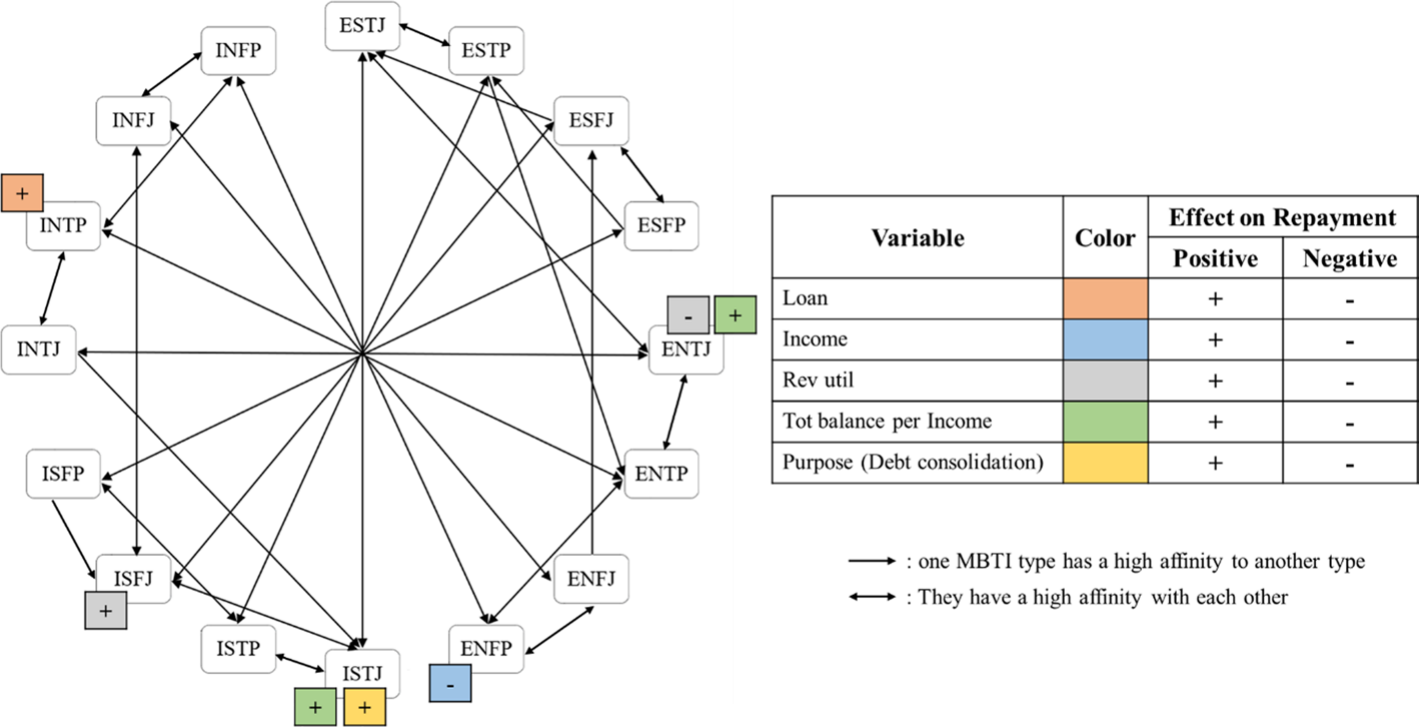
Visualization based on affinity between MBTI types and findings from LWLR

Due to the nature of the psychometric factors that influence one’s financial decision (Kuhnen and Knutson 2011), applying borrowers’ psychological propensities to online P2P lending platforms helps predict their repayment. We demonstrate that MBTI plays a role in identifying the significant effects of variables on borrower’s repayment that vary depending on their personalities in credit scoring for P2P lending. Figure 1 visualizes our findings on the affinity map to help us comprehend the results of our LWLR models. One of our findings is the reversed effects of loans and annual income on the probability of the borrowers’ repayment when they have more than a certain amount among most borrowers, regardless of their MBTI types. It clarifies the debate among many previous studies that was not clearly revealed: whether the loan and annual income positively or negatively affects the borrower’s repayment (Jiménez and Saurina 2004; Serrano-Cinca et al. 2015).

However, we found that unlike the other MBTI types, INTP borrowers pay back better as their loan amount increases. Hence, people with this type are intellectual and logical but often neglect trivial things, so the larger the loan amount, the more attentively they pay it back. Meanwhile, the higher the annual income of ENFP borrowers, the more likely they fail to pay back their loans. This result is in line with the findings that ENFP people earn more money when they have their own business.^1^ However, those self-employed lack financial knowledge known as a powerful predictor of debt (Norvilitis et al. 2006) compared with those not, thus leading to a loss of control in using the loan service (Nitani et al. 2020).

The need for psychometric factors to be introduced into the credit scoring is strengthened as we find the significant effects of some variables on repayment only for borrowers with certain MBTI types. When the revolving line usage rate is higher, borrowers with ENTJ type are less likely to repay their loan, whereas those with ISFJ type are more likely to repay their loan. Both MBTI types have a common characteristic of practicality, which is also called efficiency in their lives. However, ENTJ people seem arrogant because they believe they do not fail, whereas ISFJ people want stability and act modestly and understatedly. It causes these two types of borrowers to show opposite repayment results, depending on how much they use the revolving service. It further develops the argument of Lamdin (2008), who specified that psychometric features like one’s sentiment affect revolving-related behavior.

We also find from the borrowers with ENTJ or ISTJ types that the higher the total balance in their accounts than their annual income, the more likely they are to pay back their loan. These types of borrowers are known as being skilled in planning to achieve their goal in common. Thus, the high ratio of their balance to their annual income can be seen as having been financially well prepared for their worries about the future, so they are better able to pay back their loan if the ratio is higher. It supports an existing study arguing the tendency for cautious people to have a relatively low burden of debt and a large amount of money in their bank accounts (Dahlbäck 1991). Furthermore, borrowers with ISTJ type are more likely to pay their loan back on time when the purpose of their loan is debt consolidation, which is risky if a sufficient debt repayment plan is not supported. Given that people with this type are strategical managers who enjoy meticulously developing their long-range plans, debt consolidation can be interpreted as a method they carefully planned and selected. It supports the existing study of Bolton et al. (2011), who argued that although debt consolidation can be an effective way of borrowing for consumers who have accumulated debt or have credit problems, they must examine their newly adjusted debt punctiliously.

## Conclusion

This study proposes a psychometric credit scoring model based on the MBTI types represented using the job categories obtained from P2P lending data. The LWLR model reflects the weight based on affinity relationships among MBTI types considered to be virtual distances. Using the borrowers in the model with a personality similar to a particular MBTI type, we find that some variables have different effects on the borrowers’ repayment, depending on their MBTI types.

The results of our study shed light on the new approach to modeling an alternative credit scoring for online P2P lending. The approach used in this study suggests novel insights to create a new psychometric variable derived from job category neglected by previous studies in the P2P lending field. Through this study, we discovered significant variables not found in previous credit scoring models. We also determined how the effects of the variables on the borrowers’ repayment differ depending on their MBTI types for the job category represented by the psychometric personality. Our findings demonstrate the need for psychological reflection in credit scoring. Therefore, this study indicates that P2P lending industry must actively reflect psychometric factors in credit scoring.

This paper presents both managerial and practical implications for alternative credit scoring in P2P lending. For the relationship of the variables in P2P lending, we offer contributions to previous studies that only considered a linear effect of cash-related variables of borrowers on their repayment. Many previous studies simply expressed that the lower the loan amounts and the higher the annual income, the more likely borrowers are to repay the loans. However, this study found that the borrowers’ loan and annual income have a U-shaped and an inverted U-shaped relationship with their repayment, respectively. From a practical perspective, we note that despite the small number of MBTI samples, our LWLR model of a particular MBTI type shows higher performance than the LR model fitted to that type. It suggests that the LWLR model can produce a well-predictive credit scoring model with only few samples, including psychometric factors.

Our study left additional issues, which were our limitations. The MBTI types were inferred only from the borrowers’ respective job categories. Besides job variable, other neglected variables can be used together with other datasets to create new worthwhile variables for alternative credit scoring. Although we found that significant effects of the variables differ depending on individual personalities, such as MBTI type, in terms of practicality, techniques for improving the performance of the credit scoring models need to be further investigated due to the marginally better performance in general of the proposed alternative credit scoring. We expect more in-depth research to be conducted upon the availability of data for individual personalities, such as MBTI types. Furthermore, we look forward to the establishment of a system that allows the use of thin filers’ credit information evaluated by the P2P lending platform as a guarantee. This is because more studies suggest a new approach to alternative credit scoring in P2P lending platforms that gives thin filer the opportunity to receive a loan instead of traditional financial institutions.

## Supplementary Information

**Additional file 1**. Full results of the model for "fully paid" borrowers.

**Additional file 2**. Guidelines for R programming.

## Data Availability

The datasets generated and/or analyzed during the current study are available in the Lending Club site, https://www.lendingclub.com/investing/peer-to-peer.

## Abbreviations

DTI: Debt-to-income
LR: Logistic regression
LWLR: Locally weighted logistic regression
MBTI: Myers-Briggs type indicator
P2P: Peer-to-peer

## Footnotes

1 A survey of career outcomes among the 16 personality types from Truity Psychometrics LLC. (2015). https://www.truity.com/personality-type/ENFP/careers
