# Supplementary material for: A credit scoring model based on the Myers–Briggs type indicator in online peer-to-peer lending
Source: Financ Innov. 2022 May 3;8(1):42. doi: 10.1186/s40854-022-00347-4 (PMC9060850; doi:10.1186/s40854-022-00347-4)
Supplement: Supplementary file 1 — Additional file 1. Full results of the model for "fully paid" borrowers. [file 40854_2022_347_MOESM1_ESM.docx]

**Appendix A.**

**Table A.1.** Result of the main models for “fully paid” borrowers

|  | | LR | LWLR | | | | | | | |
| --- | --- | --- | --- | --- | --- | --- | --- | --- | --- | --- |
|  |  |  | **ENTJ** | **ENTP** | **ENFP** | **ISTJ** | **ISFJ** | **INTJ** | **INTP** | **INFP** |
| Intercept | | -0.128†  (0.069) | 0.088  (0.055) | 0.106  (0.210) | 0.058  (0.124) | 0.029  (0.041) | -0.041  (0.058) | 0.049  (0.190) | 0.092  (0.268) | 0.044  (0.202) |
| Loan | | -48.188**  (5.834) | -41.402**  (5.073) | -35.188†  (21.132) | -26.782*  (11.017) | -43.548**  (3.804) | -33.559**  (6.083) | -47.409*  (18.823) | -38.393  (23.516) | -33.996†  (19.484) |
| Loan² | | 1352.214**  (120.367) | 1377.359**  (101.545) | 1224.617**  (431.974) | 993.159**  (240.924) | 1356.752**  (75.014) | 1051.521**  (135.141) | 1494.990**  (410.588) | 1276.214*  (504.738) | 1082.683*  (432.668) |
| Income | | 9.518**  (1.394) | 4.885**  (1.118) | 4.468  (4.553) | 3.935  (2.515) | 7.476**  (0.830) | 7.720**  (1.201) | 5.658  (4.577) | 4.820  (5.799) | 4.586  (4.473) |
| Income² | | -46.388**  (6.105) | -21.748**  (4.024) | -22.077  (17.547) | -21.149*  (9.915) | -32.107**  (3.23) | -35.506**  (4.613) | -22.270  (18.444) | -21.313  (22.402) | -21.923  (17.670) |
| DTI | | -2.121**  (0.102) | -3.052**  (0.066) | -3.129**  (0.293) | -2.882**  (0.202) | -2.787**  (0.057) | -2.49**  (0.072) | -3.101**  (0.298) | -3.133**  (0.369) | -3.029**  (0.320) |
| Rev balance | | 6.682**  (0.972) | 8.367**  (0.681) | 7.375*  (2.924) | 7.280**  (1.574) | 10.475**  (0.506) | 9.544**  (0.750) | 8.506**  (2.924) | 8.434*  (3.732) | 8.961**  (3.060) |
| Rev util | | -0.193**  (0.039) | 0.100**  (0.031) | 0.115  (0.129) | 0.086  (0.067) | -0.027  (0.019) | -0.077*  (0.032) | 0.070  (0.116) | 0.078  (0.154) | 0.050  (0.132) |
| Tot balance | | -5.337**  (0.740) | -7.226**  (0.589) | -7.461**  (2.323) | -7.507**  (1.489) | -6.033**  (0.435) | -5.958**  (0.723) | -7.761**  (2.550) | -7.875**  (2.940) | -7.907**  (2.198) |
| Tot balance² | | 1.288  (0.800) | -0.447  (0.572) | 0.086  (2.261) | 0.461  (1.604) | 0.265  (0.426) | 1.047  (0.773) | -0.642  (2.709) | -0.553  (3.025) | -0.442  (2.568) |
| Cred limit | | 5.141**  (0.572) | 6.858**  (0.420) | 6.966**  (1.788) | 6.975**  (1.137) | 5.296**  (0.301) | 5.146**  (0.505) | 7.224**  (1.699) | 7.375**  (2.285) | 7.575**  (1.589) |
| Loan/Income | | -0.720**  (0.211) | -1.142**  (0.175) | -1.288†  (0.669) | -1.322**  (0.351) | -1.124**  (0.131) | -1.143**  (0.186) | -1.007†  (0.601) | -1.184  (0.793) | -1.143†  (0.610) |
| Tot balance/Income | | -0.008  (0.016) | 0.020†  (0.010) | 0.021  (0.046) | 0.019  (0.025) | 0.027**  (0.007) | 0.021  (0.013) | 0.038  (0.043) | 0.034  (0.051) | 0.025  (0.041) |
| Job | >= 10 years | 0.032*  (0.015) | 0.004  (0.010) | -0.001  (0.041) | 0.005  (0.026) | -0.002  (0.007) | 0.008  (0.011) | 0.003  (0.045) | -0.006  (0.046) | 0.007  (0.042) |
| Home | Own | -0.026  (0.027) | -0.121**  (0.019) | -0.102  (0.078) | -0.114*  (0.047) | -0.160**  (0.012) | -0.150**  (0.020) | -0.097  (0.070) | -0.124  (0.096) | -0.124  (0.076) |
|  | Rent | -0.213**  (0.020) | -0.300**  (0.014) | -0.284**  (0.060) | -0.257**  (0.037) | -0.303**  (0.009) | -0.255**  (0.017) | -0.284**  (0.055) | -0.301**  (0.071) | -0.267**  (0.055) |
| Purpose | Debt consolidation | 0.037†  (0.020) | 0.003  (0.014) | 0.024  (0.064) | 0.013  (0.035) | 0.016†  (0.009) | 0.016  (0.017) | 0.026  (0.057) | 0.021  (0.064) | 0.032  (0.065) |
|  | Credit card | 0.027  (0.026) | -0.006  (0.017) | 0.004  (0.067) | -0.019  (0.039) | 0.014  (0.012) | -0.008  (0.021) | 0.023  (0.061) | 0.024  (0.085) | 0.021  (0.071) |
| Grade | A | 1.342**  (0.029) | 1.368**  (0.019) | 1.352**  (0.074) | 1.359**  (0.042) | 1.375**  (0.015) | 1.372**  (0.024) | 1.350**  (0.088) | 1.344**  (0.093) | 1.324**  (0.089) |
|  | B | 0.816**  (0.023) | 0.823**  (0.014) | 0.810**  (0.056) | 0.801**  (0.032) | 0.836**  (0.012) | 0.803**  (0.016) | 0.814**  (0.057) | 0.808**  (0.068) | 0.795**  (0.057) |
|  | C | 0.386**  (0.021) | 0.408**  (0.013) | 0.402**  (0.063) | 0.388**  (0.029) | 0.417**  (0.010) | 0.394**  (0.018) | 0.400**  (0.060) | 0.398**  (0.059) | 0.393**  (0.064) |
| N |  | 96,996 | 202,968 | 11,606 | 31,936 | 359,604 | 138,118 | 11,630 | 8,130 | 12,290 |
| Values of the table are expressed as coefficient estimates with standard errors in parentheses.  Significance levels at † p < .10, * p < .05, and ** p < .01. | | | | | | | | | | |

**Table A.2.** Result of the remaining models for “fully paid” borrowers

|  | | LR | LWLR | | | | | | | |
| --- | --- | --- | --- | --- | --- | --- | --- | --- | --- | --- |
|  |  |  | **ESTJ** | **ESTP** | **ESFJ** | **ESFP** | **ENFJ** | **ISTP** | **ISFP** | **INFJ** |
| Intercept | | -0.128†  (0.069) | 0.073  (0.104) | 0.091  (0.143) | -0.024  (0.113) | 0.044  (0.202) | 0.058  (0.071) | -0.044  (0.134) | 0.004  (0.163) | 0.028  (0.107) |
| Loan | | -48.188**  (5.834) | -40.700**  (8.810) | -33.727*  (14.138) | -33.131**  (9.898) | -33.996†  (19.484) | -32.957**  (6.613) | -38.338**  (14.330) | -30.354*  (13.272) | -36.900**  (11.551) |
| Loan² | | 1352.214**  (120.367) | 1314.354**  (186.901) | 1179.349**  (285.375) | 1102.894**  (196.445) | 1082.683*  (432.668) | 1088.409**  (138.789) | 1230.707**  (304.320) | 1031.664**  (268.906) | 1178.670**  (228.124) |
| Income | | 9.518**  (1.394) | 6.451**  (2.007) | 5.933†  (3.106) | 7.707**  (2.311) | 4.586  (4.473) | 4.220**  (1.446) | 9.038**  (2.799) | 6.983*  (3.001) | 5.438*  (2.337) |
| Income² | | -46.388**  (6.105) | -27.886**  (7.866) | -30.624**  (11.469) | -36.987**  (8.802) | -21.923  (17.670) | -18.447**  (6.003) | -43.707**  (11.365) | -34.865**  (11.864) | -23.630**  (8.621) |
| DTI | | -2.121**  (0.102) | -2.793**  (0.136) | -2.833**  (0.231) | -2.475**  (0.146) | -3.029**  (0.320) | -2.819**  (0.111) | -2.711**  (0.236) | -2.590**  (0.238) | -2.832**  (0.175) |
| Rev balance | | 6.682**  (0.972) | 10.712**  (1.446) | 9.486**  (2.016) | 10.066**  (1.537) | 8.961**  (3.060) | 7.916**  (1.068) | 9.882**  (2.462) | 9.862**  (2.571) | 8.261**  (1.581) |
| Rev util | | -0.193**  (0.039) | -0.015  (0.053) | 0.042  (0.085) | -0.052  (0.066) | 0.050  (0.132) | 0.047  (0.042) | -0.038  (0.087) | -0.065  (0.097) | -0.016  (0.058) |
| Tot balance | | -5.337**  (0.740) | -5.418**  (1.116) | -5.934**  (1.308) | -5.801**  (1.089) | -7.907**  (2.198) | -7.002**  (0.846) | -6.819**  (2.031) | -6.395**  (1.925) | -7.221**  (1.149) |
| Tot balance² | | 1.288  (0.800) | 0.211  (1.368) | 0.849  (1.475) | 1.750  (1.192) | -0.442  (2.568) | -0.382  (0.808) | 1.670  (2.046) | 1.610  (2.156) | -0.436  (1.191) |
| Cred limit | | 5.141**  (0.572) | 4.819**  (0.707) | 5.396**  (1.066) | 4.770**  (0.832) | 7.575**  (1.589) | 6.665**  (0.588) | 5.716**  (1.506) | 5.427**  (1.308) | 6.732**  (0.863) |
| Loan/Income | | -0.720**  (0.211) | -1.177**  (0.306) | -1.339**  (0.474) | -1.254**  (0.358) | -1.143†  (0.610) | -1.152**  (0.230) | -1.195**  (0.457) | -1.368**  (0.469) | -1.055**  (0.380) |
| Tot balance/Income | | -0.008  (0.016) | 0.017  (0.022) | -0.001  (0.031) | 0.020  (0.021) | 0.025  (0.041) | 0.021  (0.015) | 0.025  (0.031) | 0.023  (0.037) | 0.032  (0.023) |
| Job | >= 10 years | 0.032*  (0.015) | -0.002  (0.018) | 0.005  (0.029) | 0.004  (0.017) | 0.007  (0.042) | 0.007  (0.015) | 0.001  (0.034) | 0.000  (0.031) | 0.009  (0.022) |
| Home | Own | -0.026  (0.027) | -0.156**  (0.032) | -0.171**  (0.044) | -0.163**  (0.035) | -0.124  (0.076) | -0.118**  (0.024) | -0.157**  (0.057) | -0.157**  (0.053) | -0.117*  (0.046) |
|  | Rent | -0.213**  (0.020) | -0.306**  (0.026) | -0.308**  (0.036) | -0.263**  (0.027) | -0.267**  (0.055) | -0.266**  (0.019) | -0.281**  (0.045) | -0.257**  (0.043) | -0.264**  (0.032) |
| Purpose | Debt consolidation | 0.037†  (0.020) | 0.006  (0.030) | -0.001  (0.036) | 0.004  (0.031) | 0.032  (0.065) | 0.007  (0.020) | 0.028  (0.042) | 0.032  (0.049) | 0.032  (0.032) |
|  | Credit card | 0.027  (0.026) | -0.015  (0.032) | -0.030  (0.046) | -0.019  (0.034) | 0.021  (0.071) | -0.017  (0.023) | 0.022  (0.056) | 0.016  (0.049) | 0.023  (0.037) |
| Grade | A | 1.342**  (0.029) | 1.406**  (0.035) | 1.422**  (0.059) | 1.388**  (0.041) | 1.324**  (0.089) | 1.346**  (0.028) | 1.367**  (0.060) | 1.362**  (0.060) | 1.316**  (0.039) |
|  | B | 0.816**  (0.023) | 0.837**  (0.030) | 0.831**  (0.034) | 0.820**  (0.023) | 0.795**  (0.057) | 0.795**  (0.020) | 0.821**  (0.042) | 0.818**  (0.040) | 0.788**  (0.029) |
|  | C | 0.386**  (0.021) | 0.411**  (0.027) | 0.407**  (0.035) | 0.404**  (0.028) | 0.393**  (0.064) | 0.383**  (0.018) | 0.417**  (0.044) | 0.401**  (0.043) | 0.393**  (0.033) |
| N | | 96,996 | 54,140 | 26,366 | 45,632 | 12,290 | 92,744 | 20,564 | 19,486 | 40,410 |
| Values of the table are expressed as coefficient estimates with standard errors in parentheses.  Significance levels at † p < .10, * p < .05, and ** p < .01. | | | | | | | | | | |
