# Supplementary material for: A credit scoring model based on the Myers–Briggs type indicator in online peer-to-peer lending
Source: Financ Innov. 2022 May 3;8(1):42. doi: 10.1186/s40854-022-00347-4 (PMC9060850; doi:10.1186/s40854-022-00347-4)
Supplement: Supplementary file 2 — Additional file 2. Guidelines for R programming. [file 40854_2022_347_MOESM2_ESM.docx]

**Supplementary Materials.**

- **R Code for LWLR**

library(caret)

library(ggplot2)

library(dplyr)

library(dplyr)

library(ROCR)

loan_MBTI_data<-read.csv("loan_data.csv") # Load

# Variable setting

loan_MBTI_data$loan_status<-as.factor(loan_MBTI_data$loan_status)

loan_MBTI_data$emp_length<-as.factor(loan_MBTI_data$emp_length)

loan_MBTI_data$home_ownership<-as.factor(loan_MBTI_data$home_ownership)

loan_MBTI_data$purpose<-as.factor(loan_MBTI_data$purpose)

loan_MBTI_data$grade<-as.factor(loan_MBTI_data$grade)

loan_MBTI_data$MBTI<-as.factor(loan_MBTI_data$MBTI)

sample_0_5y_new<-loan_MBTI_data[loan_MBTI_data$loan_status=="Charged Off",]

sample_1_5y_new<-loan_MBTI_data[loan_MBTI_data$loan_status=="Fully Paid",]

# Function to make "Oversampling-Sample" (Especially consider which "MBTI number", "how many times to oversampling")

oversampling_mbti_base<-function(mbti_number, sample_dataset, matrix_R, how_many_sample){

df_oversampling<-data.frame()

for (j in 1:16){

df_oversampling<-bind_rows(df_oversampling,

sample_dataset[sample(

which(sample_dataset$MBTI==j),

floor( ((matrix_R[mbti_number,j])*how_many_sample) ), replace=TRUE

),]

)

}

return( df_oversampling )

}

# "Affinity" score between MBTI types(from i to j) Matrix : mat_MBTI

# "Distance" between MBTI types(from i to j) Matrix: mat_MBTI_R

c1<-c(0,1,2,2,1,3,3,4,1,2,3,4,2,4,3,4)

c2<-c(1,0,3,2,2,1,3,2,2,1,4,3,3,4,4,4)

c3<-c(1,2,0,1,3,4,2,3,2,3,1,2,4,4,3,4)

c4<-c(3,1,1,0,4,3,2,2,3,2,2,1,4,4,4,3)

c5<-c(1,3,3,4,0,1,2,2,2,3,4,4,1,2,3,4)

c6<-c(3,2,4,3,1,0,2,1,4,3,4,4,2,1,3,2)

c7<-c(3,4,1,3,2,2,0,1,4,4,3,4,2,3,1,2)

c8<-c(4,3,3,2,2,1,1,0,4,4,4,3,3,2,2,1)

c9<-c(1,2,2,3,2,4,4,4,0,1,1,3,2,3,3,4)

c10<-c(2,1,4,2,4,4,3,3,1,0,2,1,4,2,3,3)

c11<-c(2,3,1,2,4,4,2,3,1,3,0,2,4,4,1,3)

c12<-c(4,2,2,1,4,4,3,2,3,1,1,0,4,3,3,2)

c13<-c(3,3,4,4,1,2,2,3,1,3,4,4,0,1,2,2)

c14<-c(4,3,4,4,2,1,3,2,3,2,4,3,1,0,2,1)

c15<-c(4,4,3,4,3,3,1,2,3,4,1,2,2,2,0,1)

c16<-c(4,4,4,3,3,2,2,1,4,3,3,2,2,1,1,0)

mat_MBTI<-cbind(c1,c2,c3,c4,c5,c6,c7,c8,c9,c10,c11,c12,c13,c14,c15,c16)

# Hyper-paramter (Write the value of each hyper-paramter: hp_D, hp_k)

hp_D = 6

hp_k = 1.5

mat_MBTI_R_1.0<-mat_MBTI

for (i in 1:16){

for(j in 1:16){

mat_MBTI_R_1.0[i,j]<-(1-((mat_MBTI_R_1.0[i,j])/hp_D)^(hp_k))

}

}

mat_MBTI_R_1.0<-t(mat_MBTI_R_1.0)

# LWLR

# Holdout Validation (7:3) & Bootstrapping (Bootstrap Sampling)

set.seed(MC_num)

rn0_5y<-createDataPartition(y=sample_0_5y_new$MBTI,p=0.7,list=F)

rn1_5y<-createDataPartition(y=sample_1_5y_new$MBTI,p=0.7,list=F)

train0_5y<-sample_0_5y_new[rn0_5y,]

test0_5y<-sample_0_5y_new[-rn0_5y,]

train1_5y<-sample_1_5y_new[rn1_5y,]

test1_5y<-sample_1_5y_new[-rn1_5y,]

train_5y<-rbind(train0_5y,train1_5y)

test_5y<-rbind(test0_5y,test1_5y)

# Example for an MBTI type (type 1)

train_5y_1 <- train_5y[which(train_5y$MBTI==1),]

test_5y_1 <- test_5y[which(test_5y$MBTI==1),]

Mean_GWLR_1_coef <- data.frame()

for (small_bootstrap in 1:100){

set.seed(small_bootstrap)

GWLR_5y_sample1_1.0<-oversampling_mbti_base(1,train0_5y,mat_MBTI_R_1.0,table(train1_5y$MBTI)[[1]]) # Charged Off

GWLR_5y_sample1_1.0<-bind_rows(GWLR_5y_sample1_1.0, oversampling_mbti_base(1,train1_5y,mat_MBTI_R_1.0,table(train1_5y$MBTI)[[1]]) ) # Fully Paid

# Number of General Sample (sample_5y) = as same as # of GWLR sample (half from train1_5y, half from train0_5y)

set.seed(small_bootstrap)

sample_5y<-bind_rows(

train1_5y[sample(

nrow(train1_5y),

nrow(train1_5y), replace=TRUE

),],

train0_5y[sample(

nrow(train0_5y),

nrow(train1_5y), replace=TRUE

),]

)

# Now, it is time to use Logistic Regression model.
